# Prune-Belly syndrome, a rare case presentation in neonatology: about one case in Yaounde, Cameroon

**DOI:** 10.11604/pamj.2020.36.102.24062

**Published:** 2020-06-17

**Authors:** Dany Hermann Ngwanou, Emmanuel Ngantchet, Georges Pius Kamsu Moyo

**Affiliations:** 1 Faculty of Medicine and Biomedical Sciences, University of Yaounde 1, Yaounde, Cameroon,

**Keywords:** Prune-Belly syndrome, urinary tract malformation, cryptorchidism

## Abstract

The Prune-Belly syndrome (PBS) is a rare pathology predominating in male infants, classically manifesting with the triad including aplasia of the abdominal wall muscles, dilatation of the urinary tract, and testicular abnormalities. We report and discuss the case of a full-term male newborn, in whom clinical examination at birth revealed abdominal wall muscle hypoplasia, cryptorchidism, urinary tract dilatation and renal failure. The diagnosis was made based on physical assessment, abdominal ultra-sonographic imaging, and blood sampling of urea and creatinine. For such cases, the recommended surgical management usually consists in a sequential surgical intervention including urinary tract reconstruction, abdominoplasty, and orchidopexy. However, these could not be practiced in due time in our patient, who died on the seventh day of life because of kidney failure. The prognosis of infants with Prune-Belly syndrome may be improved by quality antenatal follow-up, to enable the early diagnosis and preparation for prompt surgical intervention.

## Introduction

The Prune-Belly syndrome (PBS) also known as Eagle-barrett syndrome is a rare complex, congenital birth defect characterized by a triad including aplasia or hypoplasia of the abdominal wall muscles, urinary tract abnormalities and bilateral cryptorchidism [1, 2]. Male infants are most affected in close to 95% of cases [2, 3]. Other organ systems affectation may occur, including pulmonary, skeletal, gastrointestinal or cardiovascular malformations. There are forms of incomplete or Pseudo Prune-Belly syndromes (PPBS), which have been described in the literature, and are characterized by partial or unilateral hypoplasia of the abdominal wall muscles, which may be associated with ipsilateral renal, testicular or osteo-articular malformations. Such variants are described to be more common in female neonate [4, 5]. Furthermore, incomplete or PPBS appears to be more deleterious due to a higher incidence of urethral atresia, especially in females [4, 5]. Clinical forms and complications may be diverse, with stillbirths due to renal dysplasia and renal or pulmonary failure [2, 3]. This could justify the plurality of diagnostic and therapeutic procedures reported in the literature, as far as the management of PBS is concerned [1-5].

## Patient and observation

This is the case of an 8-hours-old male neonate infant who was referred from a 3^rd^ category hospital in Yaounde for a better management of congenital abdominal wall malformation. The mother of the baby was an 18-year-old primipara of Chadian nationality, without a history of chronic pathology, nor consanguinity or inbreeding. The pregnancy was poorly followed-up to a term of 37 weeks of gestation (GA) + 5 days, no infectious risk factor was found and there was not a notion of oligoamnios. Delivery was by vaginal route with cephalic presentation, and adaptation to extra-uterine life was normal with Apgar scores of 8, 10 and 10 respectively at the 1^st^, 5^th^, and 10^th^ minutes. Meconium and urine were not emitted immediately after birth. On physical exam, the baby´s hue was normal, the limbs were quadriflexed and reactive.

**Figure 1:**
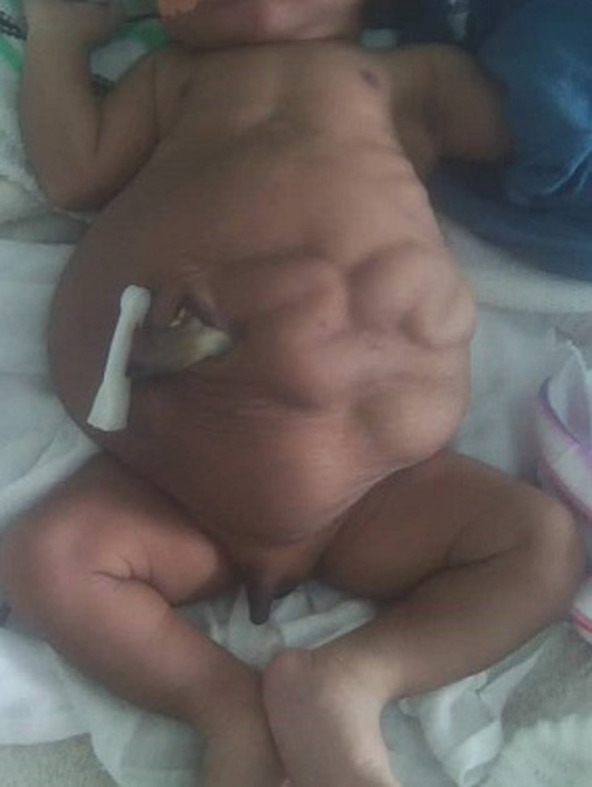
the batrachian-like spread out of the abdomen, cryptorchidism and left abdominal muscle aplasia

The vital parameters were normal as follows: temperature (T°) = 36.5°C; breathing rate (BR) = 58 Cycle per minutes; the heart rate (HR) = 148 Beats per minute; blood oxygen saturation (SPO2) = 98%. Anthropometric parameters were height = 47cm; head circumference = 35cm; the weight = 2965kg. The conjunctivae and sclera were normal, no facial dysmorphy was noted, and the nasal catheter testing for choanal atresia was negative. The cardio-pulmonary exam was normal. The abdomen was slightly distended, asymetric, with a batrachian-like spread out. The abdominal wall seemed crumpled, especially on the left flank. The intestines could be felt beneath the abdominal skin (Figure 1). The umbilical stump was fresh and normally vascularized. The osteo-articular exam was without peculiarities, with an upper segment height = 26cm, while that of the lower segment was = 21cm. The external genitalia were masculine, but the scrotum was empty, due to undescended testes. There was no sexual differentiation anomaly; the anus was present and permeable. The minimal neurological exam was normal with primitive reflexes, axial and peripheral tonus that were normal.

The paraclinical exams done comprised an abdominal ultrasound which showed features in favor of aplasia of the left antero-lateral abdominal muscles, the kidneys had normal topography, sizes (left kidney = 47mm; right kidney = 48mm), and were well-differentiated. There was however, dilatation of the ureters and pyelocaliectasis, characterizing hydro ureteronephrosis. The kidney function was altered with blood urea = 2.19 g/l, creatinine = 33.0mg/l and a creatinine clearance of 5.88ml/min. The first micturition occurred 50 hours after birth. The mother´s kidney function was rather normal. Further complementary exams such as cardiac and trans-fontanellar doppler ultrasounds, rachis and thoraco-abdominal radiography, as well as abdomino-pelvic computed tomography (CT)-scan in search of other malformations could not be done because of financial limitations.

The management consisted in the maintenance of a balanced hydroelectrolytic state, adequate postures, enteral feeding through a nasogatric tube, given the difficulty to put the baby onto the breast, and delayed digestion. The surgical management would have consisted in a sequential intervention comprising: orchidopexy, urinary tract reconstruction and abdominoplasty respectively. However, these could not be practiced in due time, because of renal failure and limited financial resources. The neonate died after 7 days of life.

## Discussion

The Prune-Belly syndrome was first described by Fröhlich in 1839 [1-3, 6]. Its estimated incidence worldwide is thought to be as low as 1 case per 40 000 live births [2]. Whereas, in Cameroon, as of now, the incidence is unknown. The real etiology of the PBS is yet to be identified, but 3 plausible hypotheses have however been suggested, including the hypothesis of prenatal urine obstruction, the embryological hypothesis over a primary mesodermic differentiation between the 6^th^ and the 10^th^ weeks of gestation which could be responsible for a defective urinary tract and abdominal musculature [2, 7]. A third hypothesis is the yolk sac theory, which proposes a dysgenesis of the yolk sac and allantois as the basis of the PBS [2, 7]. The PBS predominates in male infants with more than 95% patients being of male sex, as it was the case with our patient [8]. While the partial or pseudo PBS is more common in female infants, who rarely manifest urinary symptoms [1, 9]. Based on the male sex predominance of the PBS, a genetic influence has eventually been evoked with a possible transmission through autosomal recessive sex-linked genes [10].

The main clinical signs of the PBS are urinary tract malformations including the megavesicle, dilatation, stenosis or atresia of the urethra or ureters, polycystic kidneys, hydronephrosis, and sometimes vesico-ureteral or urethral diverticulum [1, 2, 11]. Therefore, the assessment of the kidney function seems fundamental for the prognosis [1-3, 9]. In the case of our patient with an incomplete form of PBS marked by hydroureteronephrosis, the kidney failure was fatal. Many other malformations may be associated with PBS, notably pulmonary, cardiac, skeletal, gastrointestinal or genital malformations, as described by Routh *et al*. with significant incidences of 25% for cardiovascular defects, 24% for gastrointestinal birth defects, 23% for musculo-skeletal defects, 58% for respiratory defects and 15% for genital malformations [2, 7]. Pulmonary hypoplasia is the most common associated respiratory malformation and could be responsible for varying degrees of respiratory failure which may be a major cause of precocious neonatal mortality in PBS [2-5]. Gastrointestinal malformations such as mesenteric malrotation, atresia, stenosis, volvulus, anal imperforation, splenic torsion, Hirschsprung disease and gastroschisis are quite common [2-5]. Osteo-articular birth defects such as clubfoot, hip dysplasia, vertebral malformations and scoliosis may occur as well [2-5]. Cardiovascular malformations that may be found include Fallot tetralogy and the ductus arteriosus [1, 7].

Our patient presented cryptorchidism, which occurs in almost all male neonate infant with PBS. Whereas, in female infants, genital malformations mainly involve vaginal atresia, bicornuate uterus and a urogenital sinus. In some few cases, there may be lateralization of various malformations. For example, unilateral aplasia or hypoplasia of abdominal muscles may be associated with ipsilateral defects of other organs systems such as the kidney, the testis or even osteo-articular malformations. This constitutes the Pseudo Prune-Belly syndrome (PPBS) [4, 5]. Our patient manifested an incomplete form of the PPBS with unilateral abdominal muscle defect associated with an ipsilateral ureteral dilatation and pyelocaliectasis, but the cryptorchidism was bilateral. The antenatal diagnosis relies on accurate obstetrical ultrasound which can show the typical aspect of the abdomen, and detect anomalies of the urinary system [1-3, 9]. A main difficulty in our context is the limited access to obstetrical ultrasound in a number of pregnant women, especially in rural areas where the level health care is relatively low.

Nevertheless, poorly trained echographists may miss the antenatal diagnosis, as the interpretation is operator-dependent or inaccurately revealed by outdated machines. This may lead to a delay in the diagnosis, and hence unpreparedness for the rapid management after birth. In the postnatal period, the diagnosis is made by an abdominopelvic ultrasound, which, in the best of cases should be accompanied with abdominopelvic CT-scan, trans-thoracic echography, rachis X-ray, karyotype and renal assessments [1-3, 9]. These serves a diagnostic purpose in search of other possible associated malformations and a prognostic estimation. The Karyotype helps to investigate deletion or suppression on the Hepatocyte Nuclear Factor 1-beta (HNF-1β) which may be responsible for multisystem defects in humans [1, 12]. In the case of the patient presented, the abdominopelvic ultrasound permitted to confirm the diagnosis, while the biochemical assessment by blood urea and creatinine sampling indicated a poor prognosis due to altered kidney function, as a criterion for non-operability.

The main treatment of the PBS is by surgical intervention, which consist in a series of operations including the reconstruction of the urinary tract, abdominoplasty and orchidopexy. Nevertheless, a multidisciplinary approach with efficient anesthesia, resuscitation supportive care, and the management of complications is necessary for a favorable outcome [1-3, 9, 11, 13]. For patients who are affected with mild abdominal muscles hypoplasia, postures may be helpful. However, for severe cases, surgical intervention must be discussed. Kidney transplantation may be envisaged in patients with kidney failure. Therefore, the holistic management of patients with PBS requires a multidisciplinary collaboration including the urologist, pediatrician, genetician, nephrologist, pediatric surgeon and anesthesiologist. The prognosis of PBS is often determined by the presence and severity of pulmonary hypoplasia or kidney failure. Pulmonary hypoplasia is the main cause of mortality in such patients during the neonatal period while kidney dysfunction determines in addition the long term prognosis [1-3, 9, 11].

## Conclusion

The PBS is a congenital malformation, probably underdiagnosed in our context. The diagnosis was suspected in our patient firstly on clinical examination, which revealed hypoplasia of abdominal wall muscles and cryptorchidism. Then confirmed by abdominal ultrasound. The main causes of death in this pathology being renal failure and pulmonary hypoplasia, it is important that the diagnosis is made early in order to detect potentially fatal complications in time and to realize surgical treatment quickly. We therefore recommend better monitoring of pregnancies, meticulous obstetrical ultrasound for a precise antenatal diagnosis and an improved technical platform especially in our limited-resource context, in order to reduce mortality associated with this disease.
